# Marked Body Shape Concerns in Female Patients Suffering from Eating Disorders: Relevance of a Clinical Sub-Group

**DOI:** 10.1371/journal.pone.0165232

**Published:** 2016-10-24

**Authors:** Lucie Gailledrat, Morgane Rousselet, Jean-Luc Venisse, Sylvain Lambert, Bruno Rocher, Manon Remaud, Alice Guilleux, Anne Sauvaget, Emeline Eyzop, Marie Grall-Bronnec

**Affiliations:** 1 Clinical Investigation Unit “Behavior Addictions / Complex Affective Disorders”, Addictology and Psychiatry Department, Nantes University Hospital, Hôpital Saint Jacques, 85 rue Saint Jacques, 44 093 Nantes cedex 1, France; 2 EA 4275 "Biostatistics, Pharmacoepidemiology and Subjective Measures in Health Sciences", Nantes University, Institute of Health Research IRS, 22 boulevard Bénoni Goullin, 44 200 Nantes, France; Universite de Bretagne Occidentale, FRANCE

## Abstract

Concerns about body shape and weight are core diagnostic criteria for eating disorders although intensity varies between patients. Few studies have focused on the clinical differences relative to the intensity of these concerns. Nonetheless, they might have a prognostic value. This study was aimed at identifying the characteristics associated with marked body shape concerns in patients with an eating disorder. Data was collected from a systematic and standardized clinical assessment of outpatients seeking treatment in our department for eating disorders. Only female patients, suffering from anorexia nervosa or bulimia nervosa, and with “no / mild” or “marked” body shape concerns according to the Body Shape Questionnaire, were included for the present study. We focused on sociodemographic characteristics, eating disorder characteristics, axis 1 disorders, types of attachment, self-esteem and dissociation. A multiple logistic regression was performed to identify factors related to “marked” body shape concerns. In our sample (123 participants, with a mean age of 24.3 years [range 16–61]), 56.9% had marked concerns with body shape. Marked body shape concerns were associated with a major depressive episode (OR = 100.3), the use of laxatives (OR = 49.8), a high score on the item “body dissatisfaction” of the Eating Disorders Inventory scale (OR = 1.7), a higher minimum body mass index (OR = 1.73), and a high score on the item “loss of control over behavior, thoughts and emotions” from the dissociation questionnaire (OR = 10.74). These results are consistent with previous studies, and highlight the importance of denial.

## Introduction

Eating Disorders (ED), which include in particular Anorexia Nervosa (AN), Bulimia Nervosa (BN), Binge Eating Disorder (BED) and Other Specified / Unspecified Feeding and Eating Disorders are frequent pathologies which mainly affect young women and for whom the prognosis remains guarded. According to the American Psychiatric Association [1], prevalence of AN is 0.4% and that of BN is between 0.5% and 1.5%. A study by Zipfel et al. [2] found that only half of the patients suffering from AN had recovered after 21 years. About 10% of these AN patients met all the diagnostic criteria and more than 15% had died from an ED related cause (infectious cause, hydroelectrolytic disorders, suicide). Indeed, numerous studies mention the high mortality, between 5% and 10%, associated with AN [3]. The DSM-5 maintains a 5% mortality per decade for AN and a 2% mortality per decade for BN [1].

Patients with an ED are much more concerned with their body image and weight than the rest of the population. Body image is based on two key elements: a mental picture of one’s physical body (including size, shape, and appearance), and one’s attitude toward the physical self (such as thoughts, feelings and beliefs about one’s body). It is a part of body representation, involved in conscious body-related perception and cognition. ED are usually associated with disturbances of self-perception and body size estimation [4]. Having distorted oversized body image leads the patients with an ED to body dissatisfaction, resulting in negative thoughts and feelings about their own body, and reinforces the fear of gaining weight or becoming fat. These concerns usually are intrusive and pervasive in patients with an ED. As a rule, they occupy a significant space in the clinical symptoms of ED [5], whether it be AN or BN [6]. Indeed, the DSM-5 allocates a pivotal place to concerns related to body shape and weight in the diagnosis of AN, which appear also in the definition of BN [1] (S1 Table).

Marked concerns about weight and shape are a risk factor in developing an ED [7–10]. Moreover, body image disturbances have also been shown to be a recurrent factor in the persistence of ED [11] and in relapses [12], and could thus also have a prognostic value [6]. The mechanisms underlying a distorted body image remain unclear [13]. The development of neurocognitive sciences, with an increasing number of studies assessing cognitive functioning in ED, may help to better understand the distortion of body image. To date, studies have evoked a primary impairment in the processing of spatial reference frames along with an inability to update stored representations of body shape in ED patients. These deficiencies could be related to a distortion in body image [14].

Clinical experience shows that there are various intensities of body shape concerns among patients with ED, with different evolutions. Assessing body shape concerns is a challenge, as sometimes, they are not expressed consciously [15]. This explains the change in criterion B of AN diagnosis in the firth version of DSM [1]. To the best of our knowledge, no study has yet been conducted to determine the level of body shape concerns and their correlates among patients with an ED.

The aim of our study is thus to determine whether or not the intensity of body shape concerns in ED patients is associated with specific psychopathological profiles.

## Materials and Methods

### Procedure and Ethics

Our unit is specialized in ED management and is recognized as a Reference Centre for the Region of Western France. To receive treatment in our unit, patients must be referred to us by a medical professional. We provide physical, psychological and social care in accordance with guidelines for ED management [16–18]. The care objectives of our unit are; (i) to restore patients to a healthy weight, (ii) to change core dysfunctional symptoms and attitudes related to ED (excessive concerns about body shape and weight, dietary restriction, purge and binge symptoms, etc.) and (iii) to manage all other negative features of ED. Treatment is primarily in an outpatient format, with inpatient treatment provided only if necessary.

Since September 2012, an in depth clinical assessment is carried out systematically for all new ED patients referred to our unit for treatment. The aforementioned assessment, which forms the EVALADD cohort, takes place prior to the first medical consultation and aims at highlighting the risk factors involved in ED initiation and persistence. The main criteria for inclusion in the EVALADD cohort are; an age of 16 years or older and an ED as defined by the DSM-IV (but excluding amenorrhea for AN). Patients with a cognitive impairment or with difficulties in reading and writing French are not included. All patients have a face-to-face semi-structured interview and complete self-report questionnaires. Qualified and experienced staff members performe the assessments.

The EVALADD cohort was approved by the local Research Ethics Committee (Groupe Nantais d’Ethique dans le Domaine de la Santé), by the CCTIRS (Comité Consultatif sur le Traitement de l'Information en matière de Recherche dans le domaine de la Santé) and by the CNIL (Commission Nationale de l'Informatique et des Libertés). All participants provided written informed consent (for under 18 year olds written informed consent was provided by a legal representative), in accordance with the Helsinki declaration.

### Participants

Data for our study was collected between September 2012 and June 2014, we only included patients that respected the following criteria (i) female patients, (ii) diagnosed with AN or BN and (iii) with a Body Shape Questionnaire (BSQ) score lower than or equal to 110, or above 140.

**Fig 1:**
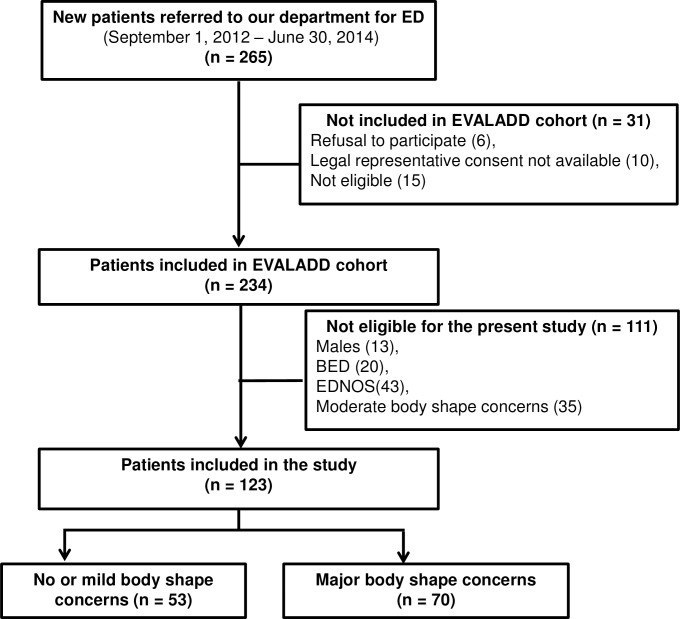
Flow chart of patient selection. (n) Number of patients included or excluded at each step of the inclusion process. (BED) Binge Eating Disorder. (EDNOS) Eating Disorders Not Otherwise Specified.

A total of 123 patients out of 234 recruits were included in the study. Fifty-three (53) had a score on the BSQ lower than or equal to 110, whereas 70 had a score on the BSQ above 140. The flow chart of patient selection is presented in Fig 1.

### Measures

Since this study was preliminary and exploratory in nature, a large range of variables was retained, all were useful in characterizing an ED and the psychopathology of the patients.

#### Socio-demographic data

The data collected concerned age, sex, marital status and professional status.

#### Eating disorder characteristics

**Structured clinical interview:** A standardized assessment of ED, specifically developed for our cohort, was performed by qualified and experienced staff members. Patients were asked about the history of their EDs (duration of ED, lowest body mass index (BMI), current ED (type of ED, current BMI), presence of associated behavior (vomiting, use of laxatives or appetite suppressants, problematic exercise, potomania, self-mutilation), personal history of physical and/or sexual abuse and family history of addictive behavior.

**Body Shape Questionnaire (BSQ):** The BSQ was conceived by Cooper et al. in 1987 [19] and the French version was validated in 2005 [20]. This self-assessment questionnaire is unidimensional and, through thirty-four items, investigates body dissatisfaction, *i*.*e*. concerns regarding weight, body shape, public embarrassment, feeling fat after eating, the banning of certain activities and the avoidance of showing one’s own body. The items reflect upon the last four weeks and answers are given on a 6-point Likert type scale ranging from “Never” to “Always”. The BSQ score provides a classification according to the intensity of body shape concerns (< 80: “no concern”; 80 to 110: “mild concerns”; 111 to 140: “moderate concerns”; > 140: “marked concerns”). The reliability of the BSQ has been reported as high (α = 0.96).

**Morgan and Russel Scale:** This structured interview examines the various ED characteristics and their repercussions during the previous six months, by way of five subscales exploring diet, menstruation, mental state, psycho-sexual functioning and socioeconomic status [21]. The average of these five scores was used as an average outcome score (Morgan and Russel total score) between 0 and 12, with 12 indicating normal functioning.

**Eating Disorder Inventory-2 (EDI-2)**: This ninety-one (91) item self-assessment questionnaire evaluates the symptomatology and the behavior associated with ED [22]. It examines 11 dimensions: “Drive for thinness”, “Bulimia”, “Body dissatisfaction”, “Ineffectiveness”, “Perfectionism”, “Interpersonal distrust”, “Interoceptive awareness”, “Maturity fears”, “Asceticism”, “Impulse regulation” and “Social insecurity”. Answers are given on a 6-point Likert type scale ranging from “Never” to “Always”. Each of these dimensions can be analyzed independently and a score is calculated for each item. Internal consistency factors for the EDI-2 dimensions are between 0.44 and 0.93.

**Contour Drawing Rating Scale (CDRS)**: This scale examines nine body shapes to which each is assigned a number, from 1 for the thinnest to 9 for the largest [23]. Patients must choose their current body shape and ideal body shape. The discrepancy between the ideal and current body shape scores is equal to the index of the body dissatisfaction score. If the resulting score is positive, body dissatisfaction is linked to a feeling of being overweight and if the score is negative, body dissatisfaction is linked to a feeling of being underweight. In the validation study, the CDRS showed a good level of reliability (r = 0.78) and validity (95.2% patients were positioned correctly).

#### Clinical characteristics

**Mini International Neuropsychiatric Interview (MINI):** The fifth version of this structured diagnostic interview allows for the main axis-I psychiatric disorders of the DSM-IV to be explored in a quick and standardized manner [24]. For the aim of this study, a French version was used [25] and only major depressive episodes and risk of suicide were considered. For major depressive episodes, the kappa coefficient (0.73), sensitivity (0.94) and specificity (0.78) were all good.

**Relationship Scales Questionnaire (RS-Q):** This 30 item self-assessment questionnaire was developed in 1991 [26] and validated in French in 2010 [27]. This questionnaire is based on the theoretical principles of Bowlby and, more specifically, on the concept of an Internal Working Model to examine four different types of attachments: “secure”, “fearful”, “preoccupied” and “dismissing”. For all items, answers were given on a 5-point Likert type scale ranging from “Not at all like me” to “just like me”. In the French translation study, cronbach’s alpha coefficient was moderate (α > 0.60) and the Intraclass Coefficients were good (> 0.75).

### Self-Esteem Scale (SES)

This self-assessment questionnaire enables a global evaluation of self-esteem based on ten items [28]. For all items, answers are given on a 4-point Likert type scale ranging from “Strongly disagree” to “Strongly agree”. Cronbach's alpha coefficient ranges from 0.77 to 0.88 and correlations from the test-retest reliability range from between 0.82 to 0.88. The global score indicates a “low self-esteem” (score <30) or a “high self-esteem” (score ≥ 30). The validation of a French version was obtained in 1990 [29].

### Dissociation Questionnaire (DIS-Q)

This 63-item questionnaire assists in the assessment of dissociative experiences according to four sub-scales: “identity confusion”, “loss of control over behavior, thoughts and emotions”, “amnesia” and “absorption” [30]. Answers are given on 5-point Likert type scale ranging from “Not at all” to “Extremely”. For each subscale an average score is calculated. Cronbach's alpha ranges from 0.69 to 0.94. The validation of a French version was obtained in 1998 [31].

#### Statistical analysis

We established two groups of patients according to BSQ scale scores: a group “no or mild concerns with body shape” (BSQ ≤ 110) and a group “marked concerns with body shape” (BSQ > 140).

A descriptive statistical analysis of the socio-demographic, clinical and ED characteristics was carried out in order to obtain means, medians and standard deviations for continuous variables, and numbers of patients, for categorical variables. An univariate analysis was performed in the following way. The links, between the socio-demographic, clinical and ED characteristics on the one hand, and the BSQ status (“low” vs “marked” concerns with body shape) on the other hand, were studied using Student tests for the quantitative variables and Chi-2 or Fisher tests for the qualitative variables [32].

Thereafter multivariate analyses (multiple logistic regression) were performed using an iterative selection procedure to select the variables that were significantly associated with the BSQ status, as assessed by the likelihood ratio test. Variables were considered as candidates for the model if they were associated to “marked concerns with body shape” in a univariate analysis with a p < 0.20. In this step, non-significant variables at 5% were removed one at a time, starting with the least significant variable (backward procedure), in order to select only the variables which provided significant information. Variables to be included in the final models were selected using a p < 0.05 criterion. The corresponding odds ratio and associated 95% confidence interval were estimated. Discrimination of the final logistic models, which describes the models ability to differentiate between “low” versus “high” BSQ scores, was assessed using the area under the Receiver Operating Characteristic (ROC) curve and the goodness-of-fit of the model was assessed using the Hosmer–Lemeshow test. The statistical analysis was carried out with SAS 9.1 and R statistical software (SAS Institute, Inc.). The conditions of validity were verified for all of the tests and models.

## Results

### Description of the complete sample

The study sample included a total of 123 patients of whom 121 had complete data.

#### Socio-demographic Data

Only women aged between 16 and 61, with a mean of 24.3 years old (SD = 9.3) were included in our sample. Most patients were single (83.7%) and unemployed (72.4%).

#### Eating Disorder Characteristics

Of the 123 patients, 46 (37.4%) had a restricting type of AN (AN-R), 28 (22.8%) had a binge eating/purging type of AN (AN-B/P) and 49 (39.8%) displayed BN. The ED had on average started 8 years before (SD = 9.15). The clinical characteristics associated with ED are detailed in Table 1.

**Table 1:** Characteristics (current or past) associated with ED (N = 123).

Characteristics	Percentages
Vomiting	61.8%
Laxatives	24.4%
Appetite suppressants	13.8%
Problematic exercise	63.3%
Potomania	25.2%
Self-mutilation	25.2%

N, number of patients; %, percentage.

The average BMI across the sample was 17.6 kg/m^2^ (SD = 3.18). Most of the patients (87) expressed body dissatisfaction due to a feeling of being overweight (62.6% with a positive CDRS score), while 34 expressed body dissatisfaction due to a feeling of being underweight (27.6% with a negative CDRS score) and 12 stated that their current body shape matched their ideal body shape (9.8% with neutral CDRS score). Amongst the 123 patients, 53 had a BSQ score below or equal to 110, and made up the first group (“no or mild body shape concerns”), whereas 70 patients had a BSQ score greater than or equal to 140, and made up the second group (“marked body shape concerns”). The highest positive scores of the EDI-2 sub-items concerned “Body dissatisfaction”, “Drive for thinness”, “Interoceptive awareness” and “Ineffectiveness”. Amongst other characteristics, physical or sexual abuse was associated with ED in respectively 6.5% and 13% of cases. Overall, 87% of patients acknowledged some prior family history of addiction (taking into account all types of addictive disorders). ED made up half (50.5%) of this type of family history.

#### Clinical Characteristics

Amongst the 123 patients, the vast majority (75.6%) reported a major depressive episode either current (30.1%) or past (45.5%). A current suicide risk was displayed in 60.2% of the sample. The psychological characteristics, as evaluated by the self-assessment questionnaires are listed in Table 2.

**Table 2:** Clinical Characteristics associated with ED (N = 123).

Variables	Mean (SD)
SES Total score	22.05 (6.05)
RSQ Secure attachment score	2.69 (0.64)
RSQ Fearful attachment score	3.39 (0.87)
RSQ Preoccupied attachment score	3.39 (0.75)
RSQ Dismissing attachment score	3.21 (0.81)
DIS-Q Total score	2.59 (0.59)
DIS-Q Confusion score	2.64 (0.84)
DIS-Q Amnesia score	1.86 (0.66)
DIS-Q Loss of control score	2.82 (0.77)
DIS-Q Absorption score	2.97 (0.73)

N, Number of patients; SD, Standard deviation; DIS-Q, Dissociation Questionnaire; RSQ, Relationship Scale Questionnaire; SES, Self-Esteem Scale.

### Comparison of the two groups of patients: univariate analysis and multiple logistic regression

Fifty-three (53) patients had a BSQ score lower than or equal to 110 and were classified in the group “no or mild concerns with body shape”. Whereas, 70 patients had a BSQ score higher than 140 and were classified in the group “marked concerns with body shape”. As shown in Table 3, an univariate analysis was performed in order to identify variables associated with marked body shape concerns.

**Table 3:** Univariate comparison of patients according to the intensity of body shape concerns (N = 123).

Variables	No or mild body shape concerns n = 53% or m (sd)	Marked body shape concerns n = 70% or m (sd)	p	Statistical tests
Age (years)	25.2 (10.7)	23.6 (8.1)	0.37	Student test
Marital status: single	83.0%	84.3%	0.45	Fischer test
Professional status: unemployed	75.5%	70.0%	0.50	Chi^2^ test
Duration of ED (years)	8.0 (10.7)	7.9 (7.8)	0.96	Student test
Type of ED: anorexia nervosa	84.9%	41.4%	< 0.01	Chi^2^ test
Current BMI (kg/m^2^)	15.9 (2.0)	18.3 (3.3)	< 0.01	Student test
Minimum BMI (kg/m^2^)	14.4 (2.0)	16.0 (2.2)	< 0.01	Student test
Vomiting (current and past)	39.6%	78.6%	< 0.01	Chi^2^ test
Use of laxatives (current and past)	7.6%	37.1%	< 0.01	Fischer test
Use of appetite suppressants (current and past)	1.9%	22.9%	< 0.01	Fischer test
Problematic exercise (current and past)	51.9%	72.1%	0.02	Chi^2^ test
Potomania (current and past)	15.1%	32.9%	0.02	Chi^2^ test
Self-mutilation (current and past)	9.4%	37.1%	<0.01	Chi^2^ test
Family history of addictive behavior	81.1%	91.4%	0.09	Chi^2^ test
Morgan & Russel total score	6.2 (1.9)	5.9 (1.7)	0.38	Student test
EDI -Drive for thinness	10.8 (5.4)	17.6 (2.3)	<0.01	Student test
EDI- Bulimia	3.4 (4.4)	12.0 (6.1)	0.02	Student test
EDI- Body dissatisfaction	8.9 (4.7)	20.7 (6.2)	<0.01	Student test
EDI- Ineffectiveness	8.7 (5.4)	16.2 (7.5)	<0.01	Student test
EDI- Perfectionism	5.6 (3.5)	7.4 (4.3)	0.01	Student test
EDI- Interpersonal distrust	5.2 (3.7)	8.6 (4.6)	<0.01	Student test
EDI- Interceptive awareness	8.5 (4.6)	16.5 (6.5)	<0.01	Student test
EDI- Maturity fears	6.3 (4.5)	7.4 (6.0)	0.23	Student test
EDI- Asceticism	5.2 (3.2)	9.8 (9.8)	<0.01	Student test
EDI- Impulse regulation	3.4 (3.8)	10.3 (7.1)	<0.01	Student test
EDI -Social insecurity	6.6 (3.6)	11.1 (4.8)	<0.01	Student test
CDRS- Body dissatisfaction	-0.51 (1.5)	3.5 (2.2)	<0.01	Student test
Major depressive episode (current and past)	52.8%	93.0%	<0.01	Chi^2^ test
Current risk of suicide	39.6%	75.7%	<0.01	Fischer test
History of physical and /or sexual abuse	5.7%	18.6%	0.06	Fischer test
RSQ -Secure attachment	14.7 (3.0)	12.5 (3.0)	<0.01	Student test
RSQ- Fearful attachment	12.5 (3.5)	14.4 (3.3)	<0.01	Student test
RSQ- Preoccupied attachment	13.4 (2.9)	13.7 (3.1)	0.63	Student test
RSQ—Dismissing attachment	15.6 (3.8)	16.4 (4.2)	0.28	Student test
SES total score	25.5 (5.2)	19.4 (5.3)	<0.01	Student test
DIS-Q Confusion	2.3 (0.7)	2.9 (0.8)	<0.01	Student test
DIS-Q Loss of control	2.4 (0.6)	3.2 (0.7)	<0.01	Student test
DIS-Q Amnesia	1.6 (0.4)	2.1 (0.7)	<0.01	Student test
DIS-Q Absorption	2.8 (0.8)	3.1 (0.7)	0.04	Student test

%, percentage; m, mean; sd, standard deviation; BMI, Body Mass Index; CDRS, Contour Drawing Rating Scale; DIS-Q, Dissociation Questionnaire; ED, Eating Disorder; EDI, Eating Disorder Inventory; RSQ, Relationship Scales Questionnaire; SES, Self-Esteem Scale.

Following to multiple logistic regression, only five variables remained independently associated with “marked concerns with body shape” (BSQ score > 140): a history of major depressive episodes (OR = 100.3); a high minimum BMI (OR = 1.7); a high score on the EDI-2 “body dissatisfaction” (OR = 1.7); the use of laxatives (OR = 49.8) and a high score on the DIS-Q “loss of control” (OR = 10.7). The results of the multiple logistic regression are detailed in Table 4.

**Table 4:** Multiple logistic regression analysis (final model): factors associated with “marked concerns with body shape” (N = 121).

Variables	OR	CI_95%_ (OR)	p-value
Major depression (current or past)	100.28	[4.36; >999.99]	0.004
Minimum BMI	1.73	[1.14; 2.46]	0.0087
Laxatives use (current or past)	49.81	[2.16; >999.99]	0.0147
EDI- Body dissatisfaction	1.68	[1.28; 2.2]	0.0002
DIS-Q—Loss of control	10.74	[2.24; 51.43]	0.003

OR, Odds ratio; CI_95%_, 95% confidence interval; BMI, Body Mass Index; DIS-Q, Dissociation Questionnaire; EDI, Eating Disorder Inventory.

The Hosmer-Lemeshow goodness-of-fit test shows that the final model is well calibrated with p = 0.96 (a high p-value indicating a good fit) [32]. The area under the ROC curve is 0.98 showing that the model discriminated well [32] between patients who had “marked concerns with body shape” (N = 70) and those who had “no or mild concerns with body shape” (N = 53).

## Discussion

Our study confirms the hypothesis of a link between the intensity of body shape concerns and specific clinical variables.

Firstly, we showed that a significant proportion of patients seeking treatment for ED had no or only mild concerns with body shape. This could be of surprise as body shape concerns are core symptoms of ED. However, this situation is not unusual and several explanations can be considered. Patients with low or normal BMI could have self-reported that they were satisfied with their current body shape whereas the interviewer could have detected that the self-evaluation was unduly influenced by body shape and weight. This raises the question of the truthfulness of patients’ responses as a whole (body shape concerns, laxative use, etc.) when using self-questionnaires. This response bias associated with the self-assessment tools could be explained by the socially desirable responses observed in adolescents and young adults with ED [33]. Furthermore, patients could admit that they are thin, while they still focus on certain body parts, especially the abdomen, buttocks or thighs [1], leading to under-evaluating the importance of body shape concerns assessed by a self-questionnaire. A recent literature review has concluded that patients with AN tend to focus on symptom-specific details, like body parts [34]. Another reason is a state of denial of any illness. Individuals with ED, in particular those suffering from AN, frequently either lack insight into or deny the problem [1]. A previous study [15] has shown that patients in a state of denial, suffering from ED, were more likely to have lower scores of body and weight concerns in self-reported measures. In our study, patients with no or mild body shape concerns were more likely to have a lower minimum BMI, fewer major depressive episodes and less laxatives use. Thus, we can presume that a part of these patients were in a more engrained state of denial of any illness and consequently had more difficulty admitting to characteristics in relation to their body shape or to the negative effects of illness (depression, loss of control). They seemingly also had more difficulty in recognizing the limits of physical health (resulting in a lower minimum BMI).

Secondly, we showed that a larger part of our sample had marked concerns with body shape, and our regression model indicated that a history of major depression, the use of laxatives, a higher minimum BMI, a high score on the “body dissatisfaction” item of the EDI scale and a high score on the “loss of control over behavior, thoughts and emotions” item on the DIS-Q were predictors of marked body shape concerns.

Indeed, we demonstrate a strong correlation between body shape concerns and major depressive episodes (current or past). These results are consistent with those available in scientific literature. For example, Hartmann et al. concluded that there is a relationship between ED and major depression [35] and Hepworth et al. concluded that a negative mood has been shown to increase body size perception [36]. Tuschen-Caffier et al. conducted an experiment, via eye-tracking, and concluded that patients with severe depression were more focused on negatively valenced body parts [37].

We also show that the use of laxatives (current or past) is closely linked to marked body shape concerns. The varying type of ED between the two groups may well have influenced our results since the use of laxatives cannot be associated with AN-R because of its very nature. One could also take a similar line with respect to the “vomiting” symptom. However, our study did not reveal any link between vomiting and marked body shape concerns, this provides material in favor of a specific and real link between marked body shape concerns and the use of laxatives. Furthermore, the association between body shape concerns and laxatives use has already been pointed out by Bryant-Waugh et al. [38]. Another study has shown that some patients with ED may abuse laxatives for different reasons or as manifestations of different forms of psychopathology [39]. Personality dimensions were not assessed in the present study, although it could be argued that our patients with marked body shape concerns displayed personality characteristics that could explain the use of laxatives.

In addition, “Body dissatisfaction” in the EDI-2 was the only EDI-2 subscale associated with marked body shape concerns. The BSQ and the EDI-2 “Body dissatisfaction” subscale investigate very similar fields relating to body shape concerns. The validation study of the BSQ showed significant correlation between the EDI-2 and the BSQ measures for this item [19]. Another study also showed a significant correlation between the BSQ and the EDI-2 “Body dissatisfaction” and the EDI-2 “Drive for thinness” subscales [40].

Moreover, the minimum BMI since the onset of illness were higher in patients with marked body shape concerns. The type of ED could also have influenced the minimum BMI, although we can also suppose that as a general rule, patients with a lower body weight exhibit a more acute state of denial and alexithymia and are therefore less in touch with their body shape concerns. To the best of our knowledge, we did not find studies about the specific influence of BMI on body concerns in a population suffering from ED. However, several studies based on adolescents and adults in the general population indicate that satisfaction with body shape and weight decreases as BMI increases, especially among females [41–45].

Finally, we have shown a link between marked body shape concerns and a high score on the DIS-Q “loss of control” subscale. The latter focuses on analyzing the loss of control over one’s own behavior, thoughts and emotions and it includes some items on controlling one’s eating habits. Thus, it would seem that feeling loss of control over one’s behavior, thoughts and emotions would rather be linked to high body shape concerns. One can presume that the type of ED does have a minimal influence on this feeling, since loss of control is more often felt with BN or AN-B/P than with AN-R, the latter being on the contrary an exercise of excessive control. As a general rule, one can also speculate that when patients are able to voice their body shape concerns, they have in fact already let go and have somewhat abandoned controlling their symptoms, at least to the point of trusting their interviewer enough to talk about them. These patients display clinical signs and behavior different from those who show no or mild body shape concerns.

An important point to highlight is that our study did not show any link between the type of ED and the intensity of body shape concerns; although the distribution of ED types was seemingly not homogeneous between the two groups of patients. Indeed, we found that a higher proportion of patients with AN-R were in the “no or mild body shape concerns” group. Conversely a higher proportion of patients with BN were in the “marked body shape concerns” group. Possibly, the sample size for each ED sub-type is insufficient to provide evidence of a link between the type of ED and the intensity of body shape concerns. Equally likely, there might not be any link between the type of ED and the intensity of body shape concerns. Other authors have used the BSQ to evaluate body shape concerns between groups of patients suffering from ED (one having only AN-R and the other a mix of BN and AN-B/P). No significant difference between the two groups of patients was reported, although they too had small samples [40].

Finally, we have not found a link between a history of sexual abuse and the intensity of body shape concerns. A history of sexual abuse during childhood is, however, a risk factor for developing body dissatisfaction [46]. Our sample was probably too small to reveal such a link since only 16 patients included had a history of sexual abuse. The prevalence of sexual abuse in the general population varies widely, according to authors, ranging from 18% [46] to 33.8% [47]. Data collection for our study began before patients were admitted into our ward. In this context, it is therefore possible that patients under-reported a history of sexual abuse.

The results must be viewed in the context of several limitations. As already partly mentioned, it is very difficult to measure a state of denial, which probably represents the major bias of this type of study and more specifically in patients suffering from AN-R. As a matter of fact, the DSM-5 recommends including the inability to gain weight as a diagnosis criterion, because many patients insist that they have no body shape concerns, yet they are paradoxically unable to reach normal weight. This item may be considered as a state of denial or as a form of disconnection. Furthermore, we have included all patients seeking treatment for their ED, whatever the duration of their disorder. Since our study is a transversal and exploratory work, these results do not enable us to qualify a scale of severity, to predict how clinical signs will evolve, nor to provide prognostic factors. However, this study did include 123 patients, and such a large number allows for a good representativity of patients seeking treatment for ED. The refusal rate in our cohort was also very low and both the interview and the self-assessment questionnaire allowed for investigating body concerns in a complementary manner.

## Conclusion

Our study confirms that it is relevant in clinical practice to investigate the intensity of body shape concerns. We show a link to the specific clinical picture and, in particular, major depressive episodes and the use of laxatives. These results should encourage clinicians to systematically evaluate body shape concerns. Furthermore, clinicians should not always be reassured by a low level of body shape concerns, that could in fact reflect a state of denial and a potentially more severe ED. We could go further by doing a longitudinal study and evaluate how patients evolve according to the intensity of their body shape concerns in order to determine whether or not they are indeed a prognostic factor. Therefore, body shape concerns can be considered as a legitimate potential therapeutic target. To this end, body dissatisfaction seems to be improved by conditioning exercises, using repeated mirror exposure focusing on attractive and unattractive body parts [48] or a brief intervention involving photographs of bodies paired with positive social feedback [49]. Furthermore, cognitive remediation could modify the impairment in body image perception.

## Supporting Information

S1 Table Diagnostic criteria for Anorexia Nervosa and Bulimia Nervosa according to American Psychiatric Association. Diagnostic and statistical manual of mental health disorders: DSM-5 5th ed. Washington DC: American Psychiatric Publishing; 2013.
(DOC)
