# Supplementary material for: Marked Body Shape Concerns in Female Patients Suffering from Eating Disorders: Relevance of a Clinical Sub-Group
Source: PLoS One. 2016 Oct 24;11(10):e0165232. doi: 10.1371/journal.pone.0165232 (PMC5077091; doi:10.1371/journal.pone.0165232)
Supplement: S1 Table — Diagnostic and statistical manual of mental health disorders: DSM-5 5th ed. Washington DC: American Psychiatric Publishing; 2013. (DOC) [file pone.0165232.s001.doc]

**S1 Table –** Diagnostic criteria for Anorexia Nervosa and Bulimia Nervosa according to the American Psychiatric Association. Diagnostic and statistical manual of mental health disorders: DSM-5 5th ed. Washington DC: American Psychiatric Publishing; 2013.

|  | **Diagnostic criteria for Anorexia nervosa** |
| --- | --- |
| A. | Restriction of energy intake relative to requirements, leading to a significantly low body weight in the context of age, sex, developmental trajectory, and physical health. *Significantly low weight* is defined as a weight that is less than minimally normal or, for children and adolescents, less than minimally expected. |
| B. | Intense fear of gaining weight or of becoming fat, or persistent behavior that interferes with weight gain, even though at a significantly low weight. |
| C. | Disturbance in the way in which one's body weight or shape is experienced, undue influence of body weight or shape on self-evaluation, or persistent lack of recognition of the seriousness of the current low body weight. |
|  | **Restricting type**: During the last three months, the individual has not engaged in recurrent episodes of binge eating or purging behavior (*i.e.*, self-induced vomiting or the misuse of laxatives, diuretics, or enemas). This subtype describes presentations in which weight loss is accomplished primarily through dieting, fasting, and/or excessive exercise. |
|  | **Binge-eating/ purging type:** During the last three months, the individual has engaged in recurrent episodes of binge eating or purging behavior (*i.e.*, self-induced vomiting or the misuse of laxatives, diuretics, or enemas). |

|  | **Diagnostic criteria for Bulimia nervosa** |
| --- | --- |
| A. | Recurrent episodes of binge eating. An episode of binge eating is characterized by both of the following:  1. Eating, in a discrete period of time (*e.g.,* within any two-hour period), an amount of food that is definitely larger than what most individuals would eat in a similar period of time and under similar circumstances.  2. A sense of lack of control over eating during the episode (*e.g.,* a feeling that one cannot stop eating or control what or how much one is eating). |
| B. | Recurrent inappropriate compensatory behaviors in order to prevent weight gain, such as self-induced vomiting; misuse of laxatives, diuretics, or other medications; fasting; or excessive exercise. |
| C. | The binge eating and inappropriate compensatory behaviors both occur, on average, at least once a week for three months. |
| D. | Self-evaluation is unduly influenced by body shape and weight. |
| E. | The disturbance does not occur exclusively during episodes of anorexia nervosa. |
